# RNA-Binding Protein Occupancy Composition Predicts Long Noncoding RNA Subcellular Localization

**DOI:** 10.3390/ijms27125593

**Published:** 2026-06-20

**Authors:** Hidenori Tani

**Affiliations:** Department of Health Pharmacy, Yokohama University of Pharmacy, Yokohama 245-0066, Kanagawa, Japan; hidenori.tani@yok.hamayaku.ac.jp

**Keywords:** long noncoding RNA, subcellular localization, RNA-binding protein, eCLIP, cross-validated prediction, nuclear retention

## Abstract

The subcellular localization of long noncoding RNAs (lncRNAs) is a central determinant of their function, yet its molecular determinants remain incompletely defined, and most existing predictors rely on the primary sequence. Because RNA-binding proteins (RBPs) are the proximal effectors of RNA compartmentalization, this study tested whether the composition of RBPs bound to a lncRNA is predictive of its nuclear or cytoplasmic localization. Enhanced crosslinking and immunoprecipitation (eCLIP) occupancy for 139 RBPs in K562 cells was integrated with the cytoplasmic–nuclear relative concentration indices (CN-RCIs) derived from matched subcellular fractionation, and localization was modeled under chromosome-grouped cross-validation with nested regularization. RBP-occupancy composition predicted localization beyond the transcript size and total binding amount (incremental cross-validated coefficient of determination, delta-R-squared = 0.17; receiver-operating-characteristic area under the curve, AUC = 0.73, a moderate-strength association; Freedman–Lane permutation, *p* = 0.005). This increment persisted (delta-R-squared = 0.12; *p* = 0.005) against an expanded baseline that additionally absorbed the transcript abundance, intron content and exon number, indicating predictive information that is not reducible to these transcript features, and the classifier was well calibrated (Brier score = 0.10; expected calibration error = 0.02). The signed coefficient profile separated RBP function systematically: factors acting in nuclear processes (splicing, 3′-end processing, and nuclear-matrix association) carried negative, nuclear-direction weights, whereas factors acting in cytoplasmic processes (translation and messenger RNA stability) carried positive, cytoplasmic-direction weights (Mann–Whitney *p* = 0.013). The profile generalized across cell lines: a K562-trained model predicted HepG2 localization (transfer AUC = 0.71 using 76 shared RBPs), and HepG2 reproduced the association independently (AUC = 0.77). The association is correlational and of moderate strength; it is presented as an interpretable, RBP-occupancy-based complement to sequence-based predictors of lncRNA localization.

## 1. Introduction

Long noncoding RNAs (lncRNAs) act through a broad repertoire of molecular mechanisms, and a common prerequisite for most of these mechanisms is the delivery of the transcript to the correct subcellular compartment. Nuclear lncRNAs scaffold chromatin and nuclear bodies and regulate transcription and splicing, whereas cytoplasmic lncRNAs modulate signaling, translation and messenger RNA (mRNA) stability [1]. Genome-wide and single-molecule measurements have shown that lncRNA localization is highly variable between transcripts and, on average, more nuclear than that of mRNAs, and that the compartment in which a lncRNA accumulates is a defining and functionally consequential property of the transcript [2,3]. Identifying what specifies lncRNA localization is therefore central to understanding lncRNA function.

The determinants of lncRNA localization are only partially understood. Primary-sequence elements and overall gene architecture contribute, because nuclear enrichment is associated with particular sequence motifs and with architectural features that disfavor efficient nuclear export [4]. These sequence signals are strong enough that machine-learning models can predict localization from the primary sequence alone, as demonstrated for lncRNAs by DeepLncRNA [5] and iLoc-lncRNA [6] and for mRNAs by RNATracker [7]. The primary sequence, however, is a distal proxy: the proximal effectors that physically retain, export or anchor a transcript are RNA-binding proteins (RBPs). Cytoplasmic lncRNAs, for example, are frequently engaged by the ribosome and the translation machinery [8], whereas nuclear retention is mediated in part by spliceosomal and nuclear-matrix factors. Despite this, localization predictors have not been constructed directly from measured RBP occupancy, and the relationship between the combination of RBPs bound to a lncRNA and its localization has not been quantified systematically or shown to generalize across cell types.

This gap is now experimentally addressable. Enhanced crosslinking and immunoprecipitation (eCLIP) provides uniformly processed, transcriptome-wide binding maps for approximately 150 RBPs in the human cell lines K562 and HepG2 [9,10], and subcellular fractionation in the same cell lines yields compartmental concentration indices (the cytoplasmic–nuclear relative concentration index, CN-RCI) computed within the lncATLAS framework [3]. Combined with a uniform lncRNA gene annotation [11], these resources allow a direct test of three questions: whether RBP-occupancy composition is predictive of lncRNA localization beyond simple transcript features; whether any such model is interpretable in terms of the known compartment-specific functions of the contributing RBPs; and whether the relationship transfers between cell lines.

This study addressed these questions for lncRNAs. Using an eCLIP occupancy of 139 RBPs in K562 cells and matched CN-RCI values, the RBP-occupancy composition was found to predict nuclear or cytoplasmic localization significantly beyond the transcript size and total binding amount, under leakage-controlled cross-validation and a conditional Freedman–Lane permutation null, and this increment persisted against an expanded baseline that also absorbed the transcript abundance, intron content and exon number. The resulting model was well calibrated and interpretable: a systematic, function-level analysis of the signed coefficients separated nuclear-process RBPs (spliceosomal, 3′-end-processing and nuclear-matrix factors) from cytoplasmic-process RBPs (translation- and stability-associated factors) without the model being given any compartment labels. The relationship also generalized across cell lines, as a K562-trained model predicted HepG2 localization and HepG2 reproduced the association independently. These results identify measured RBP occupancy as an interpretable, cross-cell-line-reproducible correlate of lncRNA localization that complements, rather than replaces, sequence-based approaches, while remaining a correlational association of moderate strength.

## 2. Results

### 2.1. A Matched Resource of RBP Occupancy and lncRNA Localization

**Figure 1:**
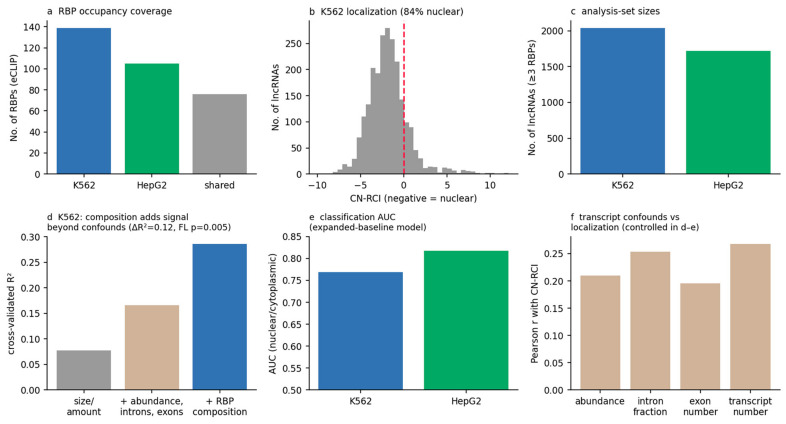
A matched resource and the predictive performance of RBP-occupancy compositions. (**a**) Number of RBPs with ENCODE eCLIP coverage in K562, HepG2 and the shared set. (**b**) Distribution of the K562 cytoplasmic–nuclear relative concentration index (CN-RCI; negative values denote nuclear localization), with the proportion of nuclear lncRNAs indicated. (**c**) Sizes of the per-cell-line analysis sets (lncRNAs bound by at least three RBPs with a localization value). (**d**) Cross-validated coefficient of determination (R-squared) in K562 for a size-and-amount baseline, an expanded baseline that additionally absorbs the transcript abundance, intron fraction and exon number, and the full model that adds the RBP-occupancy composition. (**e**) Nuclear or cytoplasmic classification area under the curve (AUC) for the expanded-baseline model in each cell line. (**f**) Pearson correlation of each transcript confounded with CN-RCI, illustrating the features controlled in panels d and e.

A matched resource was assembled for K562 cells, comprising transcriptome-wide occupancy of 139 RBPs from ENCODE eCLIP [9,10] and cytoplasmic–nuclear relative concentration indices (CN-RCIs) computed from matched subcellular fractionation within the lncATLAS framework [3] (Figure 1a–c). Strand-aware intersection of eCLIP peaks with GENCODE v44 lncRNA gene loci [11] produced a binary lncRNA-by-RBP occupancy matrix covering 7933 lncRNAs. Among lncRNAs with a CN-RCI value, 84% were nuclear (CN-RCI below zero), consistent with the known nuclear bias of the lncRNA transcriptome [2,3]. The primary analysis set comprised 2041 lncRNAs that were bound by at least three RBPs and carried a localization value; this set was similarly nuclear-skewed (87% nuclear), so imbalance-aware metrics were reported throughout.

### 2.2. RBP-Occupancy Composition Predicts Localization Beyond Transcript Size, Abundance and Architecture

A baseline model using only the transcript size (log gene length) and total binding amount (the number of bound RBPs) explained a small but significant fraction of the localization variance (cross-validated R-squared = 0.08). Adding the full RBP-occupancy composition raised the cross-validated R-squared to 0.25, an incremental delta-R-squared = 0.17 attributable to which RBPs are bound rather than how many (Figure 1d). The composition model classified nuclear versus cytoplasmic lncRNAs with an AUC of 0.73 (average precision = 0.28 against a 13% cytoplasmic prevalence; balanced accuracy = 0.67). This increment was significant under all three permutation nulls, including the conditional Freedman–Lane residual permutation (*p* = 0.005), indicating that the signal is not an artefact of the global structure or of the transcript size and amount.

To test whether this signal merely reflects correlated transcript properties, the baseline was expanded to absorb the confounds most likely to covary with occupancy: the transcript abundance (total cytosolic plus nuclear expression), intron fraction, exon number and transcript number, in addition to the length and binding amount. Each of these features was itself correlated with CN-RCI (Pearson r = 0.20 to 0.27) and with the number of bound RBPs (r = 0.34 to 0.57), and including them raised the baseline cross-validated R-squared from 0.08 to 0.17 (Figure 1d,f). Nonetheless, the RBP-occupancy composition still added a significant increment over this expanded baseline (delta-R-squared = 0.12; AUC = 0.77; Freedman–Lane *p* = 0.005), and the same was true in HepG2 (delta-R-squared = 0.14; AUC = 0.82). RBP composition therefore carries localization information that is not reducible to the transcript length, abundance, intron content or splicing complexity. The classifier was also well calibrated: out-of-fold predicted probabilities of cytoplasmic localization had a Brier score of 0.10 in K562 (0.13 in HepG2), better than a prevalence-only reference, with an expected calibration error of 0.02 in both cell lines.

### 2.3. The Signed Occupancy Profile Is Interpretable and Systematically Compartment-Coherent

**Figure 2:**
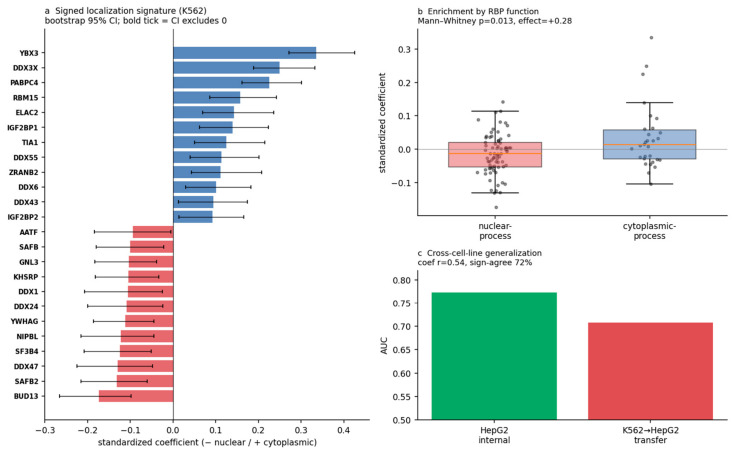
The signed occupancy profile is interpretable, systematically compartment-coherent and generalizes across cell lines. (**a**) Standardized ridge coefficients for the most nuclear-predictive (negative, red) and most cytoplasmic-predictive (positive, blue) RBPs in K562, with gene-level bootstrap 95% confidence intervals; RBPs whose confidence interval excludes zero are shown in bold. (**b**) Distribution of standardized coefficients for RBPs assigned a priori to nuclear-process versus cytoplasmic-process functional classes (Mann–Whitney p and rank-biserial effect size indicated). (**c**) HepG2-internal AUC versus the K562-to-HepG2 transfer AUC, with the Pearson correlation and sign-agreement of per-RBP coefficients fit independently in the two cell lines.

Because the model is linear, its standardized coefficients define a signed per-RBP occupancy profile (Figure 2a). Gene-level bootstrap resampling (1000 replicates) showed that the leading coefficients are stable: 27 of 139 RBP coefficients in K562 (29 of 105 in HepG2) had a 95% confidence interval excluding zero, and the median coefficient sign was reproduced in 86% of replicates. The most stable nuclear-direction RBPs (negative coefficients, 95% confidence interval excluding zero) included BUD13, SAFB2, SF3B4, DDX47 and NIPBL, and the most stable cytoplasmic-direction RBPs (positive coefficients) included DDX3X, PABPC4, IGF2BP1, RBM15 and YBX3; these are highlighted in bold in Figure 2a.

To test whether this interpretation reflects compartment-specific biology systematically rather than selected examples, every RBP in the model was assigned a priori to a functional super-class from its canonical biological-process role, independently of its fitted coefficient: a nuclear-process class (splicing, 3′-end processing, nuclear-matrix and chromatin association, ribosomal-RNA and nucleolar processing, or microprocessor and nuclear RNA decay) and a cytoplasmic-process class (translation and the ribosome, cytoplasmic mRNA stability and decay, or cytoplasmic transport). Multifunctional shuttling factors with no textbook-dominant compartment were left unclassified and excluded from the contrast. Across all classified RBPs, nuclear-process factors carried significantly more negative (nuclear-direction) coefficients than cytoplasmic-process factors (Mann–Whitney one-sided *p* = 0.013 in K562, with a rank-biserial effect size of 0.28; *p* = 0.005 in HepG2, effect size 0.40), and nuclear-process factors were enriched among the most nuclear-predictive RBPs (bottom quartile of coefficients; Fisher odds ratio = 3.0 in K562; Figure 2b). The compartment coherence of the profile is therefore a systematic property of the full coefficient distribution and is not driven by a hand-picked set of factors. Because CN-RCI is measured independently of binding, this functional partition is also non-circular in the measurement sense.

### 2.4. The Occupancy Profile Generalizes Across Cell Lines

Fitting the same pipeline independently in HepG2 (105 RBPs and 1717 lncRNAs) reproduced the association (delta-R-squared = 0.19; AUC = 0.77; Freedman–Lane *p* = 0.005). For an explicit external test, the model was restricted to the 76 RBPs shared by both cell lines, trained on K562 and applied to HepG2 (1692 HepG2 lncRNAs bound by at least three shared RBPs; 81% nuclear, comparable to 84% in K562) (Figure 2c). The K562-trained profile transferred to HepG2 with an AUC of 0.71 (average precision = 0.34; Spearman correlation = 0.43). Per-RBP coefficients fit independently in the two cell lines were positively correlated (Pearson r = 0.54) with 72% sign-agreement, and the most stable nuclear and cytoplasmic assignments were shared across cell lines. The sub-unity sign-agreement indicates partial rather than complete conservation, consistent with genuine cell-type differences in RBP function and expression as well as differences in eCLIP depth and in the lncRNAs detected in each cell line.

### 2.5. Robustness

**Figure 3:**
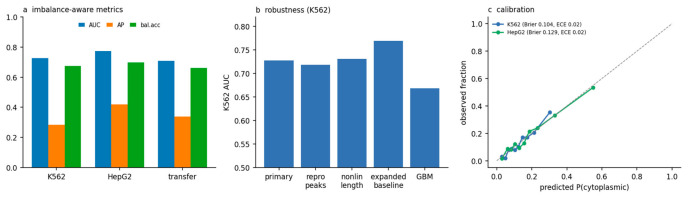
Calibration and robustness. (**a**) Imbalance-aware metrics (AUC, average precision and balanced accuracy) across K562, HepG2 and the cross-cell-line transfer. (**b**) K562 AUC for the primary model and under the reproducible-peak occupancy, a non-linear length baseline, the expanded-confound baseline, and a gradient-boosting classifier. (**c**) Reliability curves for the out-of-fold predicted probability of cytoplasmic localization in K562 and HepG2, with the Brier score and expected calibration error (ECE) indicated; the dashed line denotes perfect calibration.

The K562 result was stable under several variations (Figure 3a,b). Restricting occupancy to reproducible peaks (supported by at least two replicate files), which guards against gene-length-correlated inflation from permissive peak calling, retained the signal (AUC = 0.72; delta-R-squared = 0.14). Adding a non-linear length term to the baseline did not diminish the composition contribution (AUC = 0.73; delta-R-squared = 0.18), and the expanded-confound baseline analyzed in Section 2.2 likewise retained it. A non-linear gradient-boosting classifier did not exceed the linear model (AUC = 0.67), indicating that the localization signal is largely additive across RBPs and is well captured by the interpretable linear profile. Imbalance-aware metrics (average precision and balanced accuracy) are reported for all cohorts and variants.

## 3. Discussion

This study shows that the composition of RNA-binding proteins bound to a lncRNA, measured directly by eCLIP, is predictive of its nuclear or cytoplasmic localization, that the relationship generalizes across two cell lines, and that it is interpretable in terms of the known compartment-specific functions of the contributing RBPs. The full model improves on a size-and-amount baseline by a cross-validated delta-R-squared of approximately 0.17 (AUC approximately 0.73) under leakage-controlled cross-validation and a conditional permutation null, and the increment persists (delta-R-squared approximately 0.12) against an expanded baseline that absorbs the transcript abundance, intron content and exon number. A K562-trained model transfers to HepG2 (AUC approximately 0.71) with concordant per-RBP directions, and the classifier is well calibrated. These results identify the measured RBP occupancy as an interpretable correlate of lncRNA localization, complementary to predictors built from the primary sequence.

The strength and nature of this relationship should not be overstated. The absolute performance is moderate (AUC 0.71 to 0.77), so the model describes a probabilistic association rather than a deterministic rule that fixes the compartment of a given transcript, and a substantial fraction of localization variance remains unexplained. For this reason, the present work avoids the language of a biological “code” and instead describes a signed, interpretable RBP-occupancy profile. The analysis is correlational throughout: it establishes that the identities of the bound RBPs are statistically informative about localization, not that any individual RBP causally controls where a lncRNA accumulates. The two are linked because RBPs are the proximal effectors of compartmentalization, but the data presented here cannot, on their own, distinguish causal control from co-occurrence with other localization determinants.

This results in a biologically interpretable, non-circular readout. Because localization (CN-RCI) is measured independently of binding, the recovered profile is not circular in the measurement sense, and a systematic, function-level analysis confirms that its compartment coherence is not an artefact of the selected examples: across all classified RBPs, factors acting in nuclear processes carried nuclear-direction weights and factors acting in cytoplasmic processes carried cytoplasmic-direction weights (Section 2.3). Nuclear-predictive RBPs are dominated by spliceosomal, 3′-end-processing and nuclear-matrix or nucleolar factors (for example, BUD13, SF3B4, SAFB2, DDX47 and NIPBL among the most stable), consistent with co-transcriptional and nuclear-retention roles, whereas cytoplasmic-predictive RBPs are translation- and stability-associated (for example, DDX3X, PABPC4, IGF2BP1 and YBX3). The latter is consistent with reports that cytoplasmic lncRNAs are frequently engaged by the ribosome and translation machinery [8], and the nuclear signatures are consistent with the gene-architecture and splicing determinants of nuclear retention and selective export [4]. The readout is non-circular from a measurement standpoint but not fully independent at the level of the biological process: some strong nuclear coefficients (such as spliceosomal factors) act on the same gene-architecture features that themselves correlate with nuclear retention [4]. The profile is therefore consistent with localization reflecting which RBP machinery a transcript has engaged, although the association is correlational and does not establish causal control. Consistent with an additive, interpretable profile, a non-linear gradient-boosting model did not outperform the linear fit, indicating that the contributions of individual RBPs are largely additive and well captured by the signed linear weights.

Regarding the relationship to sequence-based predictors, sequence models predict lncRNA localization from the primary sequence [5,6] and mRNA localization through deep models [7]; the occupancy model presented here is complementary and interpretable, naming the RBPs whose binding accompanies each localization outcome. Because the primary sequence acts in part by specifying RBP binding, occupancy is the proximal molecular layer between the sequence and compartment. Integrating sequence features with measured, or computationally predicted, RBP occupancy is a natural next step that may improve both accuracy and interpretability.

There was no independent experimental validation. The present study is entirely computational and reuses publicly available datasets; it does not include perturbation experiments, RBP knockdown, orthogonal CLIP or localization assays. The RBPs identified as the strongest contributors should therefore be regarded as statistically informative correlates of localization, not as validated causal regulators. The well-calibrated, cross-cell-line-reproducible and function-coherent nature of the profile makes these factors plausible candidates for targeted perturbation, and experiments that knock down individual nuclear-predictive or cytoplasmic-predictive RBPs and measure the resulting shift in lncRNA localization would be required to establish causal control. Such experiments are an important direction for future work and are beyond the scope of this re-analysis.

Several limitations qualify these conclusions. First, the absolute performance is moderate and the lncRNA population is strongly nuclear-skewed (84% to 87%); imbalance-aware metrics and calibration are therefore reported throughout, and the AUC alone should not be over-interpreted. Second, occupancy is defined as any peak overlap across the gene locus; although a reproducible-peak variant, non-linear length controls and the expanded-confound baseline all preserved the signal, the peak-calling permissiveness and gene architecture remain potential contributors. Third, the analysis is restricted to the two cell lines (K562 and HepG2) for which both eCLIP and matched fractionation exist. Given the known cell-type specificity of RBP expression, localization mechanisms and transcriptome architecture, the conclusions should be regarded as established only for these cellular contexts; the 72% per-RBP sign-agreement, rather than near-unity, indicates partial conservation, and broader generalizability will require eCLIP and fractionation matched in additional cell types. Fourth, subcellular localization is dynamic and context-dependent, varying with the cell state, stress, differentiation, infection and disease, and the regulatory functions of RBPs are themselves highly context-dependent; for example, RBMX2 regulates intron retention and epithelial apoptosis during mycobacterial infection and has been linked to epithelial–mesenchymal-transition-associated transcriptional reprogramming and tumor progression [12,13]. A static, steady-state occupancy map of two cell lines cannot capture such dynamics, and the profile reported here should be read as a steady-state average rather than a description of regulated, condition-specific localization. Fifth, in an exploratory comparison, the same occupancy features predicted localization only numerically better than the RNA half-life on the 195 lncRNAs measured in both assays (AUC 0.61 versus 0.60; delta-R-squared 0.10 versus 0.07), and a paired bootstrap of the AUC difference was not significant (mean 0.016; 95% confidence interval −0.12 to 0.15); moreover, the only available genome-wide lncRNA half-life data are from a different cell line (HeLa BRIC-seq [14]). No localization-versus-stability dissociation is therefore claimed, and this comparison is treated only as motivation for future work with the half-life and localization matched in the same cell line. Finally, the ridge coefficients fitted under the correlated occupancy features should be read as a regularized, jointly estimated profile rather than as independent effect sizes for individual RBPs, which is why coefficient stability is reported by bootstrap confidence intervals rather than asserted per RBP.

Regarding the outlook, as eCLIP and matched fractionation data accrue in additional cell types and conditions, the occupancy profile can be tested for breadth and cell-type specificity, integrated with sequence features, and used to nominate candidate RBPs for perturbation experiments that test causal control of lncRNA localization. More broadly, the approach of predicting an independently measured RNA fate from an interpretable RBP-occupancy composition offers a template for connecting the large-scale RBP–RNA interaction maps now available to the downstream behaviors those interactions accompany.

## 4. Materials and Methods

### 4.1. Data Sources

RBP occupancy. Transcriptome-wide RBP binding was taken from ENCODE eCLIP [9,10] in two cell lines: K562 (139 RBPs, 435 peak files) and HepG2 (105 RBPs, 315 peak files), using GRCh38 peak calls downloaded from the ENCODE portal. For an RBP represented by multiple replicate peak files, peaks were pooled; a stringent reproducible variant retained a gene–RBP call only when it was supported by at least two of that RBP’s files. POSTAR3 [15] and ENCORI/starBase [16] were used during pilot exploration only.

Subcellular localization. The steady-state nuclear or cytoplasmic distribution of each gene was quantified as the cytoplasmic–nuclear relative concentration index (CN-RCI), following the lncATLAS framework [3]. The CN-RCI was computed directly from the ENCODE polyA-plus subcellular fractionation RNA-seq matched to each eCLIP cell line (K562 and HepG2; cytosol and nucleus gene quantifications). For each gene, the CN-RCI was defined as log2((mean cytosol transcripts-per-million + 0.01)/(mean nucleus transcripts-per-million + 0.01)); genes were retained when the larger of the cytosol and nucleus values was at least 1 transcript-per-million. A positive CN-RCI denotes cytoplasmic and a negative CN-RCI denotes nuclear localization.

Annotation. lncRNA gene loci were defined by GENCODE v44 [11] (long noncoding biotype).

### 4.2. Occupancy Matrix

eCLIP peaks were intersected with GENCODE lncRNA gene loci in a strand-aware manner using nested containment lists. The intersection was performed against each entire gene locus (the full genomic span, including introns and exons) rather than against the union of spliced exons. Locus-level occupancy was chosen deliberately because many RBPs, notably the spliceosomal and co-transcriptional factors that dominate the nuclear signature, bind intronic and nascent-transcript sequences that exon-level occupancy would discard; a peak overlapping any part of a lncRNA locus on the matching strand was therefore counted as occupancy. This produced a binary lncRNA-by-RBP occupancy matrix per cell line, and the number of bound RBPs per lncRNA was recorded. Analyses used lncRNAs bound by at least three RBPs and carrying a CN-RCI value.

### 4.3. Transcript Confound Covariates

To test whether the RBP-occupancy composition predicts localization beyond correlated transcript properties, an expanded baseline was constructed from features that are known or plausible confounders of both occupancy and localization. Transcript abundance was quantified as the log2 of the summed mean cytosolic and nuclear expression from the fractionation data (a proxy for steady-state abundance and transcriptional activity). Gene architecture was summarized from the GENCODE v44 annotation as the intron fraction (one minus the union exonic length divided by the gene span), the exon number (the maximum across annotated transcripts, a proxy for splicing complexity) and the transcript number. These covariates, together with the log gene length and the number of bound RBPs, defined the expanded baseline used in Section 2.2.

### 4.4. Predictive Model and Permutation Tests

The response was the CN-RCI (continuous) together with the derived nuclear or cytoplasmic class (the sign of the CN-RCI). A baseline linear model used the transcript size (log10 gene length) and total binding amount (the number of bound RBPs); the expanded baseline added the confound covariates of Section 4.3; and the full model further added the complete set of RBP-occupancy indicators (composition). Because the number of bound RBPs equals the sum of the indicators, ridge regression was used so that the full design has a unique, parameterization-invariant solution with no arbitrary reference RBP. Features were standardized, and the ridge penalty was selected by generalized cross-validation inside each outer fold, with no pre-tuning on the full response. Performance was estimated by chromosome-grouped 5-fold cross-validation, in which genes sharing a chromosome were never split across training and test folds, and was reported as the cross-validated R-squared, the ROC-AUC, the average precision and the balanced accuracy (because lncRNAs are 84% to 87% nuclear, imbalance-aware metrics were reported throughout). The incremental contribution of composition (the delta-R-squared of the full model over the relevant baseline) was assessed against three permutation nulls: global label shuffling, chromosome-blocked shuffling, and Freedman–Lane residual permutation (permuting baseline residuals within chromosome blocks), the last being the conditional null appropriate to an incremental claim.

### 4.5. Calibration

To report calibration in addition to discrimination, out-of-fold predicted probabilities of the cytoplasmic class were obtained with an L2-penalized logistic classifier under the same chromosome-grouped 5-fold cross-validation. Calibration was summarized by the Brier score, a 10-bin quantile reliability curve and the expected calibration error, with a prevalence-only model as the reference Brier score.

### 4.6. Interpretable Occupancy Profile and Coefficient Stability

Standardized ridge coefficients fitted on the full data provided the signed per-RBP occupancy profile (negative denotes nuclear and positive denotes cytoplasmic). These descriptive full-data fits are distinct from the cross-validated performance estimates. Coefficient stability was quantified by a gene-level nonparametric bootstrap (1000 replicates): for each RBP, the 95% percentile confidence interval, the sign-consistency (the fraction of replicates sharing the point-estimate sign) and the top-decile selection frequency were recorded.

### 4.7. Functional-Category Enrichment

To test whether the compartment coherence of the profile is systematic rather than driven by selected examples, every RBP in the model was assigned a priori to a functional super-class from its canonical GO biological-process and UniProt primary role, independently of its fitted coefficient: a nuclear-process class (splicing, 3′-end processing, nuclear-matrix and chromatin association, ribosomal-RNA and nucleolar processing, microprocessor and nuclear RNA decay, and noncoding-RNA biogenesis) and a cytoplasmic-process class (translation and the ribosome, cytoplasmic mRNA stability and decay, and cytoplasmic transport and granules). Genuinely multifunctional shuttling factors with no textbook-dominant compartment were left unclassified and excluded from the contrast; the full assignment is provided in Table S1 (see Supplementary Materials). The signed coefficients of the two classes were compared by a one-sided Mann–Whitney U test (with the rank-biserial effect size), and the enrichment of nuclear-process factors among the most nuclear-predictive RBPs (the bottom quartile of coefficients) was tested by a one-sided Fisher exact test.

### 4.8. Cross-Cell-Line Validation

The model was restricted to the RBP set shared by K562 and HepG2 (76 RBPs), and trained on K562 and applied to HepG2, with the scaler and model fitted on K562 only. Transfer was quantified by the ROC-AUC, average precision and Spearman correlation on HepG2, and by the Pearson and Spearman correlation and the sign-agreement of per-RBP coefficients fitted independently in the two cell lines.

### 4.9. Robustness and the Half-Life Comparison

Robustness checks re-ran the K562 model with (i) reproducible-peak occupancy, (ii) a non-linear length baseline (log10 length and its square) and (iii) a gradient-boosting classifier. As an exploratory comparison, the same K562 occupancy features were used to predict the BRIC-seq RNA half-life [14] on the lncRNAs measured in both assays; because the BRIC-seq data are from HeLa cells (a different cell line from the binding and localization data), the localization-versus-half-life difference was assessed by a paired bootstrap of the AUC difference on the matched gene set and is reported as a limitation rather than a claim.

### 4.10. Software

Analyses used Python v3.13 (Python Software Foundation, Wilmington, DE, USA) with the open-source packages pandas v2.3, NumPy v2.4, scikit-learn v1.8, SciPy v1.17 and NCLS, the GENCODE v44 annotation, and the ENCODE, POSTAR3 and ENCORI public data. All analyses are scripted (scripts/lib_loc.py and scripts/loc_*.py) and regenerate every reported number from the processed data, with a fixed random seed (0) throughout.

## 5. Conclusions

The composition of RNA-binding proteins bound to a lncRNA, measured directly by eCLIP, is a moderate-strength but reproducible and interpretable correlate of its nuclear or cytoplasmic localization. The signed occupancy profile carries predictive information beyond the transcript length, abundance and gene architecture, is well calibrated, and separates RBP function along compartment lines without being given any localization labels, and the relationship generalizes from K562 to HepG2. Because occupancy is the proximal molecular layer between the primary sequence and compartment, it provides an interpretable complement to sequence-based predictors and a principled, data-driven basis for nominating candidate RBPs for the perturbation experiments that will be required to establish the causal control of lncRNA localization. Defining the breadth, cell-type specificity and dynamics of this relationship will require eCLIP and matched subcellular fractionation in additional cell types and conditions.

## Data Availability

Publicly available datasets were analyzed in this study. RNA-binding protein occupancy was obtained from ENCODE eCLIP peak calls (K562, 139 RBPs; HepG2, 105 RBPs) and subcellular localization from ENCODE polyA-plus subcellular fractionation RNA-seq, both available at the ENCODE portal (https://www.encodeproject.org, accessed on 3 June 2026). The fractionation gene-quantification files used to compute CN-RCI were, for K562, cytosol ENCFF180ZJI, ENCFF300NAD, ENCFF268RFK, ENCFF619VQI, and ENCFF679WFF and nucleus ENCFF142LZQ, ENCFF501IXI, ENCFF492MOB, and ENCFF246WQF; and for HepG2, cytosol ENCFF644XSV, ENCFF138OAS, ENCFF781LKS, and ENCFF786CFB and nucleus ENCFF729LCL, ENCFF877KTY, ENCFF210FWG, and ENCFF349PPC. The eCLIP peak-file accessions are enumerated in the analysis scripts. The gene annotation was GENCODE v44 (https://www.gencodegenes.org, accessed on 3 June 2026). Additional public resources used were the lncATLAS framework [3], POSTAR3 [15], ENCORI/starBase [16], and published BRIC-seq RNA half-life data [14]. All analysis code (scripts/lib_loc.py and scripts/loc_*.py) regenerates every reported value from these inputs and is available from the author on reasonable request.

## Supplementary Materials

The following supporting information can be downloaded at: https://www.mdpi.com/article/10.3390/ijms27125593/s1.

## References

[1] Statello L., Guo C.J., Chen L.L., Huarte M. (2021). Gene regulation by long non-coding RNAs and its biological functions. Nat. Rev. Mol. Cell. Biol..

[2] Cabili M.N., Dunagin M.C., McClanahan P.D., Biaesch A., Padovan-Merhar O., Regev A., Rinn J.L., Raj A. (2015). Localization and abundance analysis of human lncRNAs at single-cell and single-molecule resolution. Genome Biol..

[3] Mas-Ponte D., Carlevaro-Fita J., Palumbo E., Hermoso Pulido T., Guigo R., Johnson R. (2017). LncATLAS database for subcellular localization of long noncoding RNAs. RNA.

[4] Zuckerman B., Ron M., Mikl M., Segal E., Ulitsky I. (2020). Gene architecture and sequence composition underpin selective dependency of nuclear export of long RNAs. Mol. Cell.

[5] Gudenas B.L., Wang L. (2018). Prediction of lncRNA subcellular localization with deep learning from sequence features. Sci. Rep..

[6] Su Z.D., Huang Y., Zhang Z.Y., Zhao Y.W., Wang D., Chen W., Chou K.C., Lin H. (2018). iLoc-lncRNA: Predict the subcellular location of lncRNAs by incorporating octamer composition into general PseKNC. Bioinformatics.

[7] Yan Z., Lécuyer E., Blanchette M. (2019). Prediction of mRNA subcellular localization using deep recurrent neural networks. Bioinformatics.

[8] Carlevaro-Fita J., Rahim A., Guigó R., Vardy L.A., Johnson R. (2016). Cytoplasmic long noncoding RNAs are frequently bound to and degraded at ribosomes in human cells. RNA.

[9] Van Nostrand E.L., Freese P., Pratt G.A., Wang X., Wei X., Xiao R., Blue S.M., Chen J.Y., Cody N.A.L., Dominguez D. (2020). A large-scale binding and functional map of human RNA-binding proteins. Nature.

[10] Van Nostrand E.L., Pratt G.A., Shishkin A.A., Gelboin-Burkhart C., Fang M.Y., Sundararaman B., Blue S.M., Nguyen T.B., Surka C., Elkins K. (2016). Robust transcriptome-wide discovery of RNA-binding protein binding sites with enhanced CLIP (eCLIP). Nat. Methods.

[11] Frankish A., Diekhans M., Jungreis I., Lagarde J., Loveland J.E., Mudge J.M., Sisu C., Wright J.C., Armstrong J., Barnes I. (2021). GENCODE 2021. Nucleic Acids Res..

[12] Wang C., Jiang Y., Yang Z., Xu H., Khalid A.K., Iftakhar T., Peng Y., Lu L., Zhang L., Bermudez L. (2024). Host factor *RBMX2* promotes epithelial cell apoptosis by downregulating APAF-1’s retention intron after *Mycobacterium bovis* infection. Front. Immunol..

[13] Wang C., Peng Y., Yang H., Jiang Y., Khalid A.K., Zhang K., Xie S., Bermudez L., Yang Y., Zhang L. (2025). *RBMX2* links *Mycobacterium bovis* infection to epithelial-mesenchymal transition and lung cancer progression. eLife.

[14] Tani H., Mizutani R., Salam K.A., Tano K., Ijiri K., Wakamatsu A., Isogai T., Suzuki Y., Akimitsu N. (2012). Genome-wide determination of RNA stability reveals hundreds of short-lived noncoding transcripts in mammals. Genome Res..

[15] Zhao W., Zhang S., Zhu Y., Xi X., Bao P., Ma Z., Kapral T.H., Chen S., Zagrovic B., Yang Y.T. (2022). POSTAR3: An updated platform for exploring post-transcriptional regulation coordinated by RNA-binding proteins. Nucleic Acids Res..

[16] Li J.H., Liu S., Zhou H., Qu L.H., Yang J.H. (2014). starBase v2.0: Decoding miRNA-ceRNA, miRNA-ncRNA and protein-RNA interaction networks from large-scale CLIP-Seq data. Nucleic Acids Res..

